# Supplementary material for: Causal Relationship between Aging and Anorexia Nervosa: A White-Matter-Microstructure-Mediated Mendelian Randomization Analysis
Source: Biomedicines. 2024 Aug 16;12(8):1874. doi: 10.3390/biomedicines12081874 (PMC11351342; doi:10.3390/biomedicines12081874)
Supplement: Supplementary file 1 [file biomedicines-12-01874-s001.zip › Supplementary Figure/Figure S8/Leaveoneout plot of MR analysis for TL to ubm-b-1819.pdf]

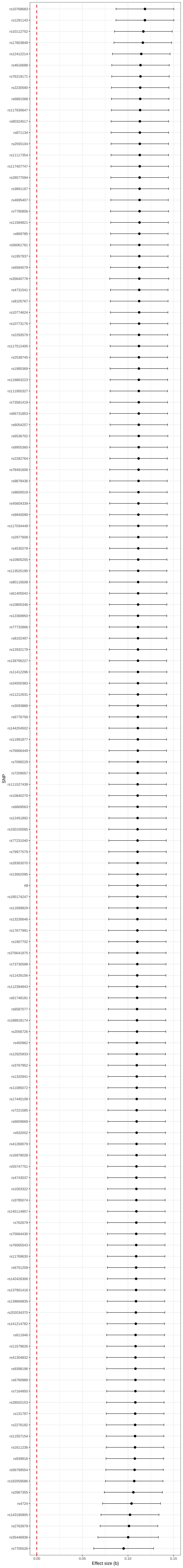
rs10768683
rs1291143
rs10112752
rs17803849
rs12412214
rs4616688
rs76219171
rs2230590
rs6881568
rs117630647
rs80324517
rs871134
rs2555104
rs11117354
rs117407747
rs28577594
rs3891167
rs4695407
rs7790856
rs11584821
rs809785
rs56061761
rs1957937
rs6584579
rs35640778
rs4731541
rs8105767
rs10774624
rs10773176
rs2293579
rs117512405
rs2538745
rs1985369
rs116863223
rs111950327
rs73581419
rs66731853
rs6054257
rs6536702
rs9955360
rs2282764
rs78491606
rs9878436
rs9600019
rs45604339
rs9940099
rs117034449
rs2977608
rs4530278
rs10905255
rs113525195
rs80116508
rs61405042
rs10805346
rs12369950
rs77732866
rs8102497
rs12932179
rs139795227
rs11412296
rs34550383
rs11212631
rs3093888
rs6776756
rs144204502
rs11991877
rs76666449
rs7099229
rs7209057
rs111527438
rs10840270
rs6669563
rs12451892
rs150150565
rs77231040
rs79977579
rs28363070
rs13062095
All
rs185174247
rs11699829
rs13230646
rs17677991
rs1907702
rs376641875
rs73730598
rs11426156
rs112394943
rs61748181
rs6587577
rs188918174
rs2056726
rs450962
rs12925933
rs3767952
rs1332941
rs11085072
rs17445108
rs7221585
rs6659669
rs932002
rs41269079
rs16978028
rs55747751
rs4743037
rs1003322
rs3785074
rs145114957
rs762679
rs75684430
rs76065543
rs11769630
rs6751209
rs142426306
rs137901416
rs139669835
rs202034370
rs141214782
rs611646
rs11579626
rs41304832
rs9398196
rs6790988
rs7164950
rs28502153
rs131797
rs2276182
rs11557154
rs1611236
rs939916
rs56799554
rs182059586
rs2967355
rs4724
rs143190905
rs2763979
rs35446936
rs7705526
SNP
0.00
0.05
0.10
0.15
Effect size (b)
